# The Antioxidant Activity and Induction of Apoptotic Cell Death by *Musa paradisiaca* and *Trigona* sp. Honey Jelly in ORL115 and ORL188 Cells

**DOI:** 10.21315/mjms2023.30.1.7

**Published:** 2023-02-28

**Authors:** Mohd Nur Nasyriq Anuar, Muhammad Ibrahim, Nor Hafizah Zakaria, Solachuddin Jauhari Arief Ichwan, Muhammad Lokman Md Isa, Nur Aizura Mat Alewi, Abdullah Hagar, Fadzilah Adibah Abdul Majid

**Affiliations:** 1 Department of Nutrition Sciences, Kulliyyah of Allied Health Sciences, International Islamic University Malaysia, Pahang, Malaysia; 2 Institute of Marine Biotechnology, Universiti Malaysia Terengganu, Kuala Nerus, Terengganu, Malaysia; 3 Dentistry Programme, PAPRSB Institute of Health Sciences, Universiti Brunei Darussalam, Gadong, Brunei Darussalam; 4 Department of Basic Medical Sciences for Nursing, Kulliyyah of Nursing, International Islamic University Malaysia, Pahang, Malaysia

**Keywords:** antioxidant, apoptosis, head and neck squamous cell carcinoma, Musa paradisiaca, stingless bee

## Abstract

**Background:**

Head and neck cancer patients usually need nutritional support due to difficulties in swallowing and chewing. Therefore, this study aimed to formulate *Musa paradisiaca* and *Trigona* sp. honey jelly (MTJ) as a convenient functional food.

**Methods:**

The antioxidant properties were analysed using 2,2′-diphenyl-1 picrylhydrazyl (DPPH), ferric reducing antioxidant potential (FRAP) and 2,2′-azinodi 3-ethylbenthiazolinesulfonate (ABTS) assays. Cytotoxicity was assayed using the 3-(4,5-dimethylthiazol-2-yl)-2,5-diphenyl tetrazolium bromide (MTT) test and the induction of apoptosis was observed via caspase-3/7 activity assay. The identification of phenolic compounds was done via ultra-high-performance-liquid chromatography coupled to mass spectrometer (UHPLC-MS/MS).

**Results:**

The antioxidant analysis exhibited: the half inhibitory concentration (IC_50_) of DPPH inhibition, 54.10 (SD = 4.51) μg/mL; the FRAP value, 30.07 (SD = 0.93) mM TEQ/100 g; and the ABTS value, 131.79 (SD = 8.73) mg TEQ/100 g. Cinnamic acid was the most abundant phenolic compound, followed by maleic acid and salicylic acid. The IC_50_ for ORL115 and ORL188 were 35.51 mg/mL and 43.54 mg/mL, respectively. The cells became rounded and dissymmetrical which reduced in number and size. The apoptotic cell death in ORL115 and ORL188 was deduced as caspase-3/7 activities that significantly increased (*P* < 0.05).

**Conclusion:**

The study evidenced that the antioxidant activity of MTJ could influence the induction of apoptosis in ORL115 and ORL188 in future investigations and verifications.

## Introduction

Head and neck squamous cell carcinoma (HNSCC) is one of the most aggressive cancers that occurs at the mucosal epithelium of the oral cavity such as the hard palate, lips, anterior tongue and nasopharynx (1). The treatments usually consist of surgical resection, followed by chemoradiation or chemotherapy. These treatments usually have side effects such as dysphagia, voice dysfunction and odynophagia (2). Patients become nutritionally deficient because these treatments affect their appetite, made them nauseous and cause vomiting (3). Therefore, healthy eating habits are crucial for cancer patients and they need to be nutritionally supported by eating the right foods that are rich in carbohydrates, fat, vitamins, minerals, protein and water (4).

*Musa paradisiaca* (*M. paradisiaca*) or locally known as *pisang awak* is frequently utilised as a supplement in foods and beverages. *M. paradisiaca* contains starch, which is the main component in energy-producing carbohydrates (5). It also contains a substantial amount of minerals (calcium, sodium, magnesium and potassium), moisture, vitamins and protein (6). It is widely documented to possess antioxidant, anticancer and antimicrobial properties. The bioactive compounds in *Musa* sp. are reported to have antiproliferative properties against several cancer cell lines such as skin, oral, colorectal, breast, cervical, hepatic, oesophageal and prostate (7).

*Trigona* sp. or locally known as *kelulut* is a stingless bee species that can be found in Malaysian rainforests. Besides kelulut honey, other types of honey in Malaysia include *gelam*, *acacia*, *tualang* and *nenas*. Generally, honey is well-known for its therapeutic potentials such as antioxidant, anticancer, antimicrobial, antidiabetic, anti-inflammatory and antihypertension properties (8–10). Honey from *Trigona* sp. is widely documented to contain more antioxidants than other types of honey (11). It is also cited to possess a cytotoxicity effect on several cancer cell lines (12). Most of the biological activities of honey are attributed to its main components, namely phenolic acids and flavonoids. It is reported that phenolic acids are the most abundant antioxidant compounds in *Trigona* sp. honey. A study revealed that *Trigona* sp. honey contains higher total phenolic contents than *gelam, tualang* and *nenas* honey (13).

Despite their benefits, little is known about the combination of *Musa* sp. and *Trigona* sp. as a convenient functional food in cancer treatment. A previous study found that the combination of honey and *Musa* sp. in honey bread had improved the nutritional and functional values of the product (14). *Trigona* sp. showed significant antioxidant activity when combined with underutilised fruit, *Baccaurea angulata* or *belimbing dayak*, through the response surface methodology via central composite design (15). Another study found that honey has a better antioxidant profile when combined with apple and passion fruit beverages (16). These works of literature indicated that the combinations of honey with fruits have the potential for the development of functional foods, especially for cancer treatment.

Functional food is defined as food fortified with additional ingredients to improve the health characteristics of a product (17). The functional jelly approach was specifically designed and targeted cancer patients because the consumption of functional food may improve the quality of life without altering the eating habit and reduce the severe effects of cancer treatment. Therefore, this study aimed to examine the antioxidant properties, cytotoxic effect and apoptotic induction of *M. paradisiaca* and *Trigona* sp. honey jelly (MTJ) on the human squamous epithelial cell lines, namely ORL115 and ORL118.

## Methods

### M. paradisiaca and Trigona sp. Honey

The bananas (*M. paradisiaca*) were purchased from a local market in Kuantan, Pahang, Malaysia. The fruit was dried and processed into powder form that was kept in an airtight container before analysis. The researcher collected the *Trigona* sp. honey sample from a local beekeeper situated in Ketereh, Kelantan, Malaysia (14, 15).

### M. paradisiaca and Trigona sp. Honey Jelly Formulation

The preparation of MTJ was done in two phases (15). About 200 g of coarse grain sugar (Gula Prai, Malaysia) and 200 g of glucose syrup (Healthy Baker, Malaysia) were mixed and heated together with 50 g of water in the first phase. Then, 40 g of gelatine (Halagel, Malaysia) was added to 110 g of water and heated again to 60 °C in the second phase. Next, 20 g of *M. paradisiaca* powder and 20 g of *Trigona* sp. honey were added to the mixture. The mixture was stirred gently before being poured into a synthetic rubber mould.

### M. paradisiaca and Trigona sp. Honey Jelly Extraction

MTJ (5 g) was suspended in 25 mL of 80% (v/v) methanol (Merck, Germany) and heated at 55 °C (15). The mixture was degassed and sonicated for 30 min. A syringe filter (0.2 μm) was used to filter the mixture. The final extract was stored at 4 °C prior to analysis.

### 2,2′-diphenyl-1 picrylhydrazyl Scavenging Assay

The 2,2′-diphenyl-1 picrylhydrazyl (DPPH) scavenging assay was analysed in triplicates (18). Initially, the methanolic DDPH solution (Sigma-Aldrich, USA) was mixed with jelly extracts (1,000 μg/mL–7.8125 μg/mL). Ascorbic acid standards (Sigma-Aldrich, USA) with concentrations of 1,000 mg/mL–0.5 mg/mL were also prepared. The readings of absorbance were taken at 517 nm using a UV/VIS-spectrophotometer (Schott UVLine 9400, USA). The DPPH scavenging assay activity was calculated using the following equation:


$$\text{DPPH radical-scavenging activity }(\%)=[({\text{A}}_{\text{o}}-{\text{A}}_{1})/{\text{A}}_{\text{o}}]\times 100$$

where A_o_ is the absorbance of the control and A_1_ is the absorbance of the sample. Then, the percentage of inhibition was plotted against concentration. From the graph, IC_50_ was calculated.

### Ferric Reducing Antioxidant Potential Assay

The ferric reducing antioxidant potential (FRAP) reagent was prepared by mixing 0.3 M of acetate buffer (Sigma-Aldrich, USA), 10 mM of 2,4,6-tripyridyl-s-triazine (TPTZ) (Sigma-Aldrich, USA) and 10 mM of ferric (III) chloride (Sigma-Aldrich, USA) in a 10:1:1 ratio, respectively (19). The solution was kept warm at 37 °C in a water bath. The absorbance of the FRAP solution as the blank was measured at 593 nm using a UV/VIS-spectrophotometer (Schott UVLine 9400, USA). Then, the reading of MTJ extract was obtained at the same wavelength. 8-tetramethyl-chroman-2-carboxylic acid (Trolox) (Sigma-Aldrich, USA) was used as the standard for the construction of the calibration curve. The result was expressed in millimolar Trolox equivalence per 100 g of MTJ (mM TEQ/100g). This assay was done in triplicates.

### 2,2′-azino-di-3-ethylbenthiazolinesulfonate Assay

The 2,2′-azino-di-3-ethylbenthiazolinesulfonate (ABTS) assay was performed using ABTS cation radical decolourisation (20). The ABTS aqueous solution (7 mM) was mixed with potassium persulphate (140 mM) (Sigma-Aldrich, USA) and left to stand at room temperature for 16 h. The mixture was diluted with methanol until it reached the absorbance value of 0.70 (SD = 0.02) at 734 nm. The jelly extract (20 μL) was mixed with 100 μL of ABTS working solution and incubated in the dark for 6 min before the measurement. The readings were measured in triplicates at 734 nm against the blank using a UV/VIS-spectrophotometer (Schott UVLine 9400, USA). A standard calibration curve was plotted using 0.625 μg/mL–10 μg/mL of Trolox (*r^2^* *=* 0.7513). The result was expressed in mg of Trolox equivalence per 100 g of MTJ (mg TEQ/100g).

### Identification of Phenolic Compounds

This study used an ultra-high-performance liquid chromatography coupled to mass spectrometer (UHPLC-MS/MS) (Perkin Elmer, USA). Targeted multiple reaction monitoring (MRM) for quantitation and qualification (negative electrospray ionisation mode) was employed as the detection mode. The compounds namely cinnamic acid, gallic acid, caffeic acid, coumaric acid, kaempferol, p hydroxybenzoic acid, maleic acid, rutin, salicylic acid, quercetin, sinapic acid and vanillic acid were used as the standards. Before being injected into LC-MS/MS, the extract and standard (5 ng/mL–200 ng/mL) were filtered through 0.45 μM nylon filters. Phenomenex Synergy RP C18, 100A, 100 mm × 3 uM × 2.0 mm was used as the column. The mobile phase consisted of water with 0.1% formic acid (solvent A) and acetonitrile with 0.1% formic acid (solvent B) (21).

### Cell Lines and Culture

Cancer Research Malaysia (CRM) provided the human oral squamous epithelial cell lines, ORL115 and ORL188, for this study. ORL115 and ORL188 cells were maintained in Dulbecco’s modified eagle medium (DMEM) (Himedia, China) with the supplementations of 10% (v/v) of foetal bovine serum (FBS) (Himedia, China) and 1% of streptomycin (100 μg/mL) (Gibco, USA). The cell cultures were maintained at 37 °C in a humidified atmosphere containing 5 % carbon dioxide (CO_2_) using a CO_2_ incubator (22).

### The MTT Assay

The cells were seeded into a 96-well plate (SPL, Korea) containing 6 × 10^4^ cells with a 100 μL complete medium (23). The cells were treated with a serum-free medium containing MTJ in a series of concentrations (1.25 g/mL–20 g/mL) and incubated at 37 °C in a humidified atmosphere containing 5% of CO_2_ for 24 h, 48 h and 72 h. The 20 μL of 3-(4,5-dimethylthiazol-2-yl)-2,5-diphenyl tetrazolium bromide (MTT) solution (5 mg/mL) in phosphate saline buffer (PBS) (Merck, Germany) was added to each well and the cells were incubated at 37 °C for 4 h. The media was aspirated carefully and 200 μL of dimethyl sulfoxide (DMSO) (Merck, Germany) was added to dissolve the formation of formazan crystal in the wells. The plate was re-wrapped in aluminium foil and shaken using a shaker at room temperature for 17 min. The absorbance readings were measured in triplicates at 570 nm using a microplate reader (Infinite M200 PRO, Switzerland). GraphPad Prism 7 software was used to determine the IC_50_ and SPSS statistical software version 16 was used for statistical analysis. The value of *P* < 0.05 was considered significant following the student’s *t-*test and Tukey’s HSD post hoc analysis.

### Morphological Observation

The morphological changes of the apoptotic cells were observed at 24 h, 48 h and 72 h under a light microscope (EVOS^TM^ XL Core Imaging System, USA) at 10× magnification (24).

### Caspase-Glo^®^ 3/7 Activity Assay

Caspase-Glo^®^ 3/7 activity assay (Promega, USA) was used to measure caspase-3 and −7 activities in ORL115 and ORL188 after the treatment with MTJ. The cells were treated after reaching 60% of confluency. Then, 70 μL of Caspase-Glo® 3/7 reagent was added to the 96-well plate. The contents of wells were mixed using a plate shaker at 300 rpm–500 rpm for 30 s. The plate was incubated at room temperature for 30 s before the luminescence of each well containing the samples, for blank or control, was measured using a luminometer (Luminoskan ascent, Thermo scientific, USA). The caspase activity was detected as the fold change of expression (25).

## Results

### Antioxidant Properties

For the DPPH assay, the antioxidant activity was determined in terms of inhibition concentration (IC_50_). The MTJ showed 54.10 (SD = 4.51) μg/mL of IC_50_ value. For FRAP and ABTS assays, the MTJ exhibited 30.07 (SD = 0.93) mM TEQ/100 g and 131.79 (SD = 8.73) mg TEQ/100 g, respectively.

### Identification of Phenolic Compounds

Table 1 shows 10 phenolic compounds with cinnamic acid exerting the highest value, followed by maleic acid and salicylic acid. Kaempferol showed the least abundant concentration, while sinapic acid and vanillic acid were not detected.

### Cytotoxicity Study

**Figure 1:**
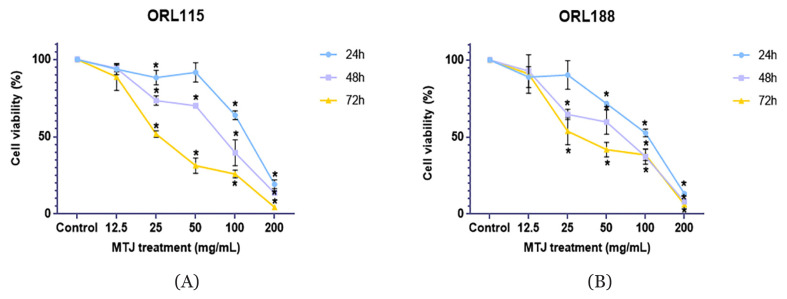
Dose-response curve on the effect of MTJ on ORL115 (A) and ORL188 (B) cell viability at 24 h, 48 h and 72 h of exposure. Values are expressed as mean (standard deviation) performed in triplicates measurement (*n* = 3). *P < 0.0001 by the comparison with the respective control

Different concentrations of MTJ were compared (12.5 mg/mL, 25.0 mg/mL, 50 mg/mL, 100 mg/mL and 200 mg/mL) at 24 h, 48 h and 72 h of exposure (Table 2). Untreated cells served as controls, which exhibited 100% of cell viability for both ORL115 and ORL188. The MTJ treatment significantly inhibited the proliferation of ORL115 and ORL188 cells in a concentration and time-dependent manner. The IC_50_ for ORL115 and ORL188 were also presented in Table 2. Figure 1 supports these data, which show a significant reduction of cell viability for both cancer cell lines after 24 h, 48 h and 72 h in a concentration and time-dependent manner.

### Morphological Observation

**Figure 2:**
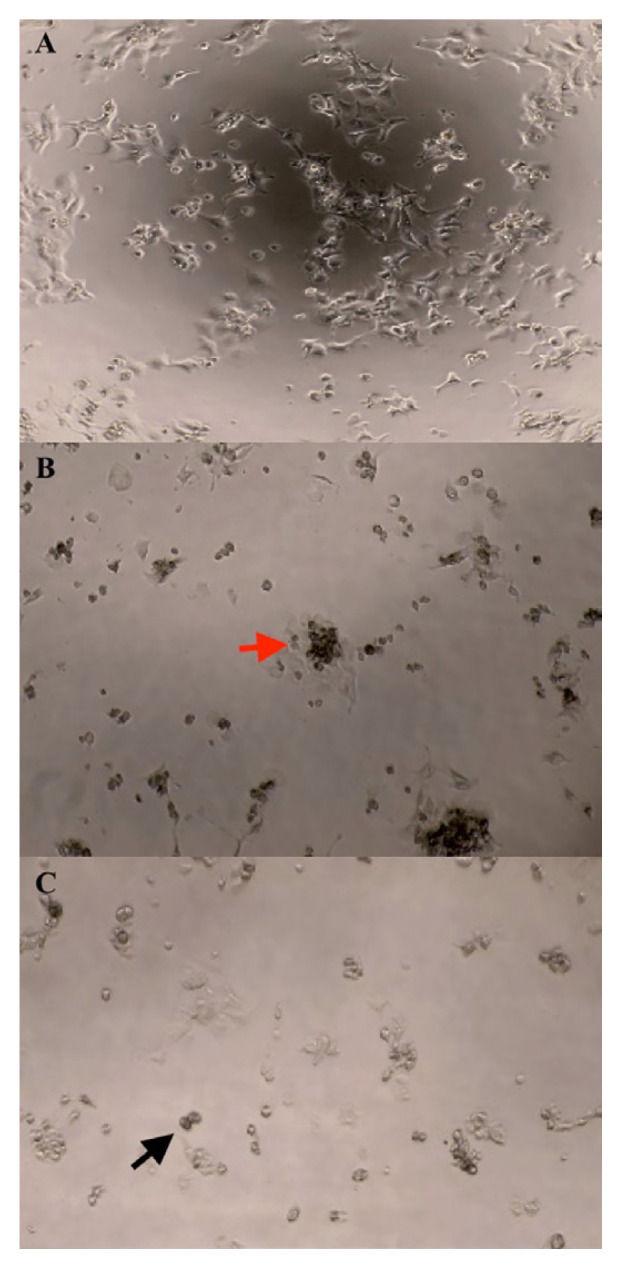
Morphological changes of ORL115 treated with MTJ at 48 h under light microscope (10×); A (Control), B (100 mg/mL) and C (200 mg/mL). Black arrow = rounded shape; red arrow = clumping cells

**Figure 3:**
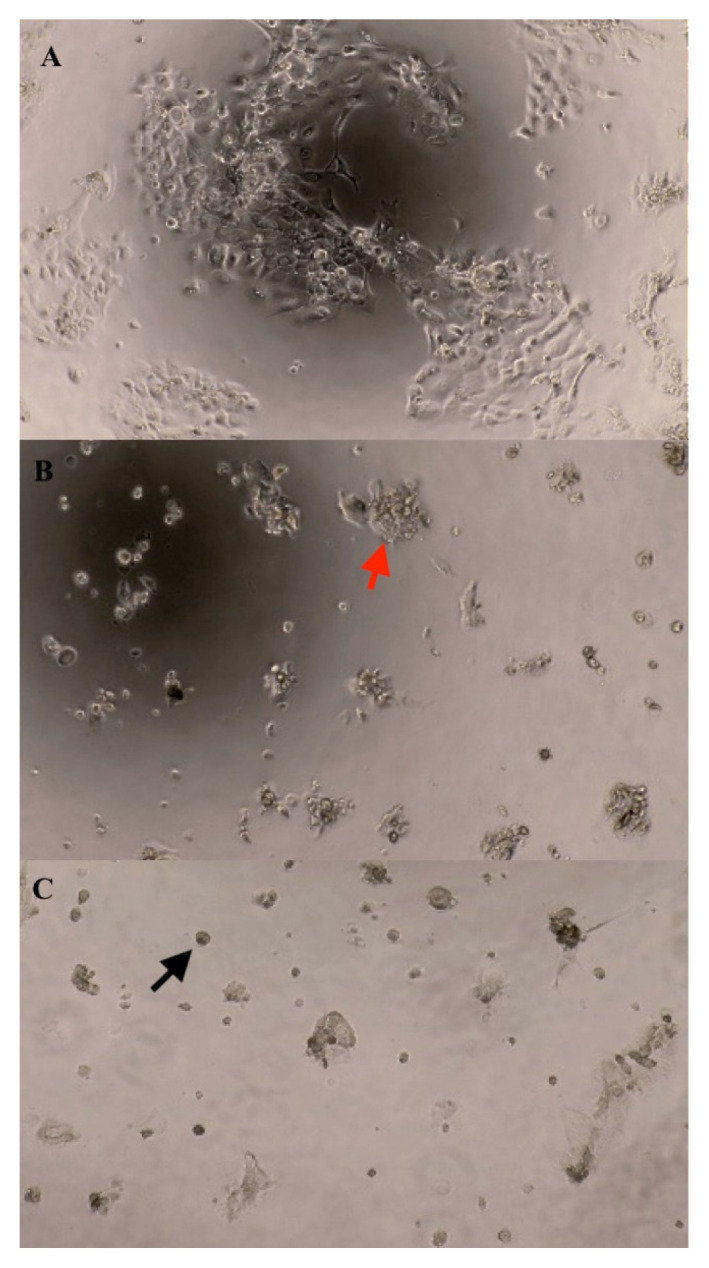
Morphological changes of ORL188 treated with MTJ at 48 h under light microscope (10×); A (Control), B (100 mg/mL) and C (200 mg/mL). Black arrow = rounded shape; red arrow = clumping cells

Through the microscopic morphological observation, MTJ caused distinctive alterations in the cell morphology for both ORL115 and ORL188. Figures 2 and 3 show that the cells have a polygonal shape for the control treatment, which is the normal shape of squamous epithelial cells. However, the cells transformed into a rounded shape and became dissymmetrical after the MTJ treatment. The cells were observed to grow in small clumps with their neighbouring cells. Floating cells also appeared, and the size and number of cells decreased following the MTJ treatment concentration of 100 mg/mL and 200 mg/mL.

### Caspase-Glo^®^ 3/7 Activity Assay

**Figure 4:**
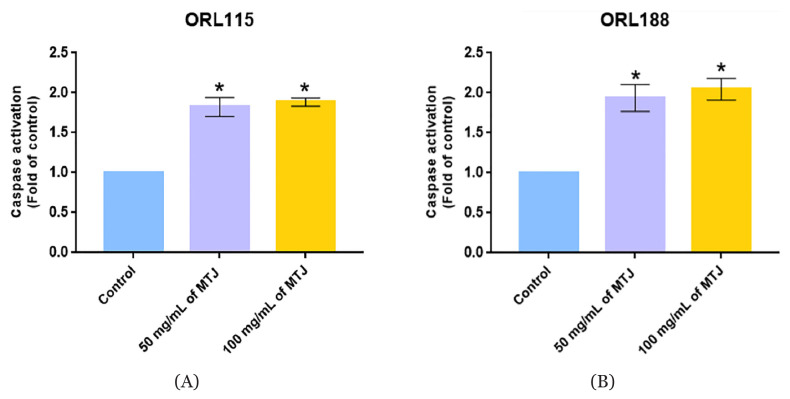
Caspase-Glo® 3/7 activity assay for ORL115 (A) and ORL188 (B). Values are expressed as mean (standard deviation). *P < 0.001 by the comparison to respective control

The activation of Caspase-Glo^®^ 3/7 assay was evaluated in ORL115 and ORL188 to establish the cell death pathway induced by MTJ as illustrated in Figure 4. The caspase activity significantly increased (*P* < 0.001) several folds in a concentration-dependent manner in ORL115 and ORL188 in relative to the control. However, there were no significant differences (*P* > 0.05) in the caspase expression between 50 mg/mL and 100 mg/mL of MTJ treatment for both cells.

## Discussion

The formulation of MTJ aimed to ease the difficult experiences of oral cancer patients in consuming foods orally. The soft structure of MTJ can aid the feeding and swallowing activities. Besides that, the therapeutic effect of MTJ such as antioxidants and anticancer could play a role in cancer treatment. The phenolic compounds have been reported as part of the antioxidant constituents, which are abundant in natural resources (26). Many studies documented the antioxidant activities of natural products via various mechanisms, including enzymatic or non-enzymatic (6, 27, 28). However, this is the first study to report the antioxidant activity and its cytotoxic effect of a formulated jelly with the combination of *M. paradisiaca* and *Trigona* sp. honey as a functional food.

DPPH, FRAP and ABTS assays were conducted to elucidate the antioxidant mechanisms of the developed MTJ. In this study, MTJ showed a lower DPPH-radical scavenging activity (IC_50_) when compared to IC_50_ (1000 μg/mL–1500 μg/mL) of the jellies formulated from banana peels (28). The lower IC_50_ indicated the high power of antioxidant capacity. In another study, the formulated *M. acuminata* Colla peel jellies, including commercial fruit jellies, revealed the IC_50_ value for DPPH inhibition that varied from 3,110 μg/mL to 4,280 μg/mL, which was also higher than MTJ (29).

A previous study demonstrated that the FRAP analysis of pomegranate jelly ranging from 5.65 mM to 18 mM TEQ/100g (30) was also lower than MTJ. A similar trend was reported when a quince fruit jam showed an FRAP value of 1.349 (3.35) mM Trolox/100 g (31). ABTS assay is a method to determine the antioxidant activity. The present study used this method and found that the MTJ activity was higher compared to the jelly formulated from pomegranate, which ranged from 2.46 mg to 3.69 mg TEQ/100g (32). Findings from DDPH, FRAP and ABTS assays proved that MTJ has a high radical scavenging activity.

Based on the UPHPLC-MS/MS result, three major phenolic acids were abundant in MTJ, namely cinnamic acid, maleic acid and salicylic acid. It is assumed that the high concentration of cinnamic acid in MTJ may possess a high capacity to induce apoptosis and subsequently, inhibit the aggressive proliferation of cancer cells. This statement is supported by a previous study, which revealed that the cinnamic acid treatment for 24 h, 48 h and 72 h significantly inhibited the growth of leukaemia cells (33). Cinnamic acid was also reported to show an apoptotic effect against human nasopharyngeal carcinoma cells as the number of apoptotic cells increased in comparison to the control group after being treated with cinnamic acid at 24 h (34).

As the concentration (12.5 mg/mL–200 mg/mL) and exposure time (24 h–72 h) increased, the trend of the proliferation inhibition for both cell lines also increased. The interactions of cinnamic acid, salicylic acid, maleic acid, rutin and kaempferol might produce the synergistic effects of MTJ. The antiproliferative properties of these compounds have been reported to induce apoptosis (28). During apoptosis, the cells display typical morphological features such as the formation of clumping cells, the transformation of cells into a rounded shape, the appearance of floating cells, and the reduction of cell number (35). These features were observed in both ORL115 and ORL188 that were treated with MTJ and compared with the untreated cells. Additionally, the IC_50_ values became lower as the incubation time was lengthened from 24 h to 72 h. It could be presumed that as the cancer cells were exposed to the samples for a longer period, lower concentrations of samples were required to achieve a reduced cancer cell viability of 50%.

In general, the caspase test was conducted to quantify the concentration of the expressed caspase, specifically caspase-3 and −7, which could be detected when the apoptotic effects were stimulated (36). Caspase is a proteolytic enzyme that plays a main role in apoptosis by cleaving the specific proteins in the nucleus and cytoplasm (37). The apoptotic effects of banana and honey have been widely cited by many authors (3, 38). However, this is the first report of apoptotic induction of banana and honey formulated jelly on head and neck cancer cells. This result could be further validated using a caspase-3 and −7 genes knockout study.

## Conclusion

Overall, the findings from the study evidenced that MTJ has promising cytotoxic and apoptosis effects on head and neck squamous cells. The cell proliferation inhibition and apoptosis phenomenon might be modulated by the presence of antioxidant compounds in MTJ, specifically cinnamic acid, maleic acid, and salicylic acid. In addition, further analysis of the exact mechanism involved in apoptosis needs to be verified. The findings of this study will provide new insights for future cancer treatment, especially from the nutrition perspectives and views.

## Figures and Tables

**Table 1:** Quantitative analysis of the phenolic compounds (ng/mL) of MTJ

Standards	Retention time (min)	Regressin equation	*r^2^ value	MTJ (ng/mL)
Caffeic acid	2.63	y = 7.5e + 003x	0.9932	0.54
Cinnamic acid	4.06	y = 569x	0.996	43.42
Coumaric acid	3.03	y = 1.81e + 004x	0.9917	0.28
Gallic acid	1.41	y = 4.24e + 003x	0.9977	4.02
Kaempferol	4.72	y = 798x	0.999	0.18
Maleic acid	0.995	y = 7.41e + 003x	0.9907	18.07
P hydroxybenzoic acid	3.31	y = 2.94e + 004x	0.9983	1.28
Quercetin	4.33	y = 5.16e + 003x	0.9957	1.07
Rutin	3.39	y = 3.94e + 003x	0.9989	0.72
Salicylic acid	2.19	y = 5e + 003x	0.9961	10.89
Sinapic acid	ND	y = 1.88e + 003x	0.9984	ND
Vanillic acid	ND	y = 542x	0.9997	ND

* *r*^2^ value is the R-squared value obtained from the standard calibration curve using series of concentration of reference standards.

ND = not detected

**Table 2:** Cell viability (%) for ORL115 and ORL188

Concentration of MTJ (mg/mL)	24 h		48 h		72 h	
		
	ORL115	ORL188	ORL115	ORL188	ORL115	ORL188
Control	100.0 (0.0)^A,X^	100.0 (0.0)^A,X^	100.0 (0.0)^A,X^	100.0 (0.0)^A,X^	100.0 (0.0)^A,X^	100.0 (0.0)^A,X^
12.5	93.6 ± (3.5)^AB,X^	88.9 (6.7)^A,X^	94.2 (2.1)^A,X^	92.8 (0.1)^A,X^	88.6 (8.8)^A,X^	90.9 (12.5)^A,X^
25.0	88.3 (4.7)^B,X^	90.3 (9.2)^A,X^	73.4 (3.0)^B,Y^	64.7 (3.3)^B,Y^	51.8 (2.1)^B,Z^	53.7 (8.8)^B,Z^
50.0	91.6 (6.3)^AB,X^	71.6 (0.8)^B,X^	70.00 (0.9)^B,Y^	59.7 (7.9)^B,Y^	31.30 (5.0)^C,Z^	41.8 (4.7)^B,Z^
100	63.90 (2.8)^C,X^	52.7 (2.5)^C,X^	39.70 (8.4)^C,Y^	37.3 (5.0)^C,Y^	25.90 (2.5)^C,Z^	38.4 (3.7)^B,Y^
200	19.20 (2.90)^D,X^	13.3 (0.2)^D,X^	13.6 (1.2)^D,Y^	8.2 (1.1)^D,X^	4.41 (1.10)^D,Y^	6.2 (0.1)^C,X^
lC_50_ (mg/mL)	105.8	90.6	65.1	56.9	35.5	43.5

Notes: Values are expressed in mean (SD), n = 3. Different superscript letters (A,B,C,D) within the columns for each concentration indicates significantly difference (Tukey’s multiple comparisons test, *P* < 0.05). Different superscript letters (X,Y,Z) within the rows for each time interval indicates significant difference (Tukey’s multiple comparisons test, *P* < 0.05)
